# Effectiveness of massed cognitive processing therapy for posttraumatic stress disorder: A retrospective analysis

**DOI:** 10.1002/jts.70045

**Published:** 2026-01-19

**Authors:** Lara Baez, Jennifer Huberty, Jacqlyn Yourell, Courtney Jewell, Eric Lin, Debra Kaysen, Lashauna Cutts, Sofia Noori, Isobel Rosenthal, Kathleen Chard

**Affiliations:** ^1^ Fit Minded Inc. Phoenix Arizona USA; ^2^ Nema Health Branford Connecticut USA; ^3^ VA Palo Alto California USA; ^4^ Cincinnati VA Medical Center Cincinnati Ohio USA

## Abstract

Trauma exposure can lead to posttraumatic stress disorder (PTSD), a condition with significant individual and societal costs. Yet, access to evidence‐based PTSD treatment, such as cognitive processing therapy (CPT), remains limited. Delivering CPT on an accelerated (i.e., *massed*) schedule via telehealth could increase access while preserving effectiveness. This study aimed to evaluate changes in PTSD, anxiety, and depressive symptoms over the course of virtual massed CPT delivered by a commercial virtual trauma clinic. The sample included adult patients with clinician‑diagnosed PTSD who completed 3 or more telehealth CPT sessions per week. PTSD, anxiety, and depressive symptoms were assessed at intake, discharge, and 30 and 90 days postdischarge. Paired *t* tests compared symptoms from intake to discharge; linear mixed‑effects models examined symptom trajectories over time. Analyses included 148 patients. Large effect sizes were observed for improvements in PTSD (Δ*M* = 39.00), *d* = 2.94; anxiety (Δ*M* = 8.90), *d* = 1.79; and depressive symptoms (Δ*M* = 8.23), *d* = 1.49, from intake to discharge. Symptom improvements for PTSD and depression were maintained at 30 and 90 days, whereas anxiety remained significantly improved from baseline at both time points but showed modest rebound between 30 and 90 days. Virtual massed CPT produced rapid, very large, and durable reductions in PTSD, depression, and anxiety. These findings support virtual massed CPT as a feasible option to expand access to evidence‑based PTSD care in community settings.

Almost 90% of the U.S. adult population experiences at least one traumatic event (e.g., serious injury, sexual violence) in their lifetime, and around 6% of individuals who have been exposed to a traumatic event develop posttraumatic stress disorder (PTSD; Kilpatrick et al., 2013). Empirical studies suggest that approximately 3.6% of U.S. adults experience PTSD per year, with estimates reaching up to 9.1%, and lifetime prevalence estimates range from 3.4% to 26.9% (Schein et al., 2021). PTSD is defined by the onset of symptoms across four clusters—intrusion, avoidance, negative alterations in mood and cognition, and alterations in arousal and reactivity—that persist for at least one month (American Psychiatric Association, 2022). PTSD is associated with an increased risk of cardiovascular disease, stroke, depression, and substance use disorders (Edmondson & Cohen, 2013; Najavits et al., 2020), and costs the United States over $232,000,000,000 (USD) in direct health care costs (e.g., medical visits), direct non–health care costs (e.g., disability), and indirect costs (e.g., productivity loss; Davis et al., 2022). Evidence‐based PTSD treatment is cost‐effective compared to treatment as usual or no treatment for adults with PTSD (von der Warth et al., 2020), but access to effective, evidence‐based PTSD treatment in community settings remains limited.

Cognitive processing therapy (CPT) is one of the gold‐standard treatments for PTSD and is recommended as a first‐line treatment in the clinical practice guidelines of multiple U.S. organizations (American Psychological Association, 2025; Resick, LoSavio, et al., 2024; U.S. Department of Veterans Affairs [VA]/Department of Defense [DoD], 2023). CPT is a cognitive behavioral therapy that addresses the maladaptive thought patterns and related emotions that patients develop following exposure to a traumatic event (Resick, Monson, & Chard, 2024). In CPT, therapists help patients address unhelpful beliefs about themselves, others, and the world that patients develop after a traumatic event (e.g., “The world is a dangerous place”; Resick, LoSavio, et al., 2024). Patients attend, on average, 12 weekly therapy sessions, with a typical range of seven to 15 sessions, and are expected to complete therapy practice assignments on their own between sessions (e.g., cognitive worksheets and behavioral assignments). CPT is effective across various settings and populations and has been shown to reduce PTSD symptoms in both the short and long terms (Resick, LoSavio, et al., 2024). Meta‐analytic findings also point strongly to CPT's efficacy, with effect sizes ranging from 1.24 to 1.63 for posttreatment PTSD symptom reductions (Asmundson et al., 2019; Watts et al., 2013).

Although CPT is highly effective in treating PTSD, patient engagement remains a perennial challenge for all trauma‐focused evidence‐based treatments, with dropout rates upwards of 30% (Alpert et al., 2020). Maximizing treatment completion is important because patients who complete treatment are more likely to improve their PTSD symptoms (Holmes et al., 2019), and attending more sessions is associated with higher levels of symptom improvement and higher remission rates (Szafranski et al., 2017). Several factors contribute to dropout, including patient avoidance and slow early progress that can affect motivation (Ragsdale et al., 2020; Sherrill et al., 2022). Practical considerations that may impact CPT engagement include scheduling difficulties, transportation issues, and work conflicts (Kantor et al., 2017), highlighting the importance of innovative delivery models that help patients complete treatment and obtain the full therapeutic benefit of CPT (Schottenbauer et al., 2008).

Massed (or “accelerated”) CPT was developed to increase treatment engagement and retention and meet the needs of patients who want treatment delivered on an accelerated timeframe (Ragsdale et al., 2020). In massed CPT, patients meet for sessions daily or up to three times per week for 1–4 weeks. The theoretical basis for massed CPT suggests that consolidating sessions may reduce avoidance behaviors between sessions, maintain therapeutic momentum, reduce external distractions that interfere with treatment progress, and promote early symptom relief that reinforces continued engagement over time (Gutner, Gallagher, et al., 2016; Gutner, Suvak, et al., 2016). Several studies have demonstrated that massed CPT is as effective as traditional CPT (Goetter et al., 2021; Wachen et al., 2024). Dropout rates in massed CPT trials are low, with approximately 10% of participants discontinuing treatment (Goetter et al., 2021). However, to date, studies that have evaluated massed CPT have been conducted in veteran and/or military service populations or small community samples (Goetter et al., 2021; Held et al., 2022, 2024).

Teletherapy may improve retention in PTSD treatment by reducing barriers such as travel time and cost (Jones et al., 2020). Several studies have shown that CPT delivered virtually is feasible, acceptable, and as effective as in‐person care (Peterson et al., 2022). In a pilot study of 1‐week virtual massed CPT in which treatment sessions occurred twice daily for 5 days, treatment completion rates were high (95.8%), and patients experienced large reductions in PTSD symptoms (*d* = 2.55; Held et al., 2022). Similarly, a study of veterans receiving telehealth‐delivered massed PTSD treatment reported 81.8% completion rates with large reductions in PTSD symptoms (*d* = 1.48; Verdi et al., 2025). Virtually delivered massed CPT may address multiple barriers simultaneously, which could be especially valuable to patients who may not otherwise have access to evidence‐based PTSD care. Commercial teletherapy platforms have sought to meet the growing demand for mental health services, including PTSD treatment (Morland et al., 2020; Owusu et al., 2025), and they represent a promising avenue for broadening access to evidence‐based interventions. Commercial platforms function as large, digitally organized care systems. They often combine extensive provider networks with mobile application (app)–based interfaces, scheduling tools, and other digital supports that structure how care is accessed and delivered (Achtyes et al., 2023). Given the diversity of digital service models and delivery contexts, ongoing research is needed to establish the effectiveness of treatment delivery across large‐scale commercial platforms.

The current study aimed to evaluate changes in PTSD, anxiety, and depressive symptoms over the course of virtual massed CPT delivered by a commercial virtual trauma clinic and up to 3 months postdischarge. We aimed to (a) test whether PTSD, anxiety, and depressive symptom scores significantly improved from intake to discharge and assess the magnitude of these effects; (b) examine symptom trajectories across intake, discharge, and 30‐ and 90‐days postdischarge from the intensive phase of treatment (i.e., CPT protocol); and (c) determine whether symptom improvements observed at discharge were maintained at 30‐ and 90‐days postdischarge. We hypothesized that PTSD, anxiety, and depressive symptoms would significantly decrease from intake to discharge and that these gains would be maintained across both 30‐ and 90‐day follow‐up assessments.

## METHOD

### Participants and procedure

This study is a retrospective analysis of patient data from a virtual telehealth program offering massed CPT for PTSD. The program operated across multiple U.S. states and provided rapid access to trauma‐focused care via a HIPAA‐compliant telehealth platform. Patients could self‐refer or be referred by health care providers and typically began treatment within days of completing a brief intake evaluation. Services were reimbursable through major commercial insurers, workers’ compensation, or self‐pay arrangements, with sliding‐scale options available to improve financial accessibility.

Patients in this study underwent CPT multiple times per week (i.e., at least three sessions and up to five sessions), for an average of 3–4 weeks via secure videoconferencing. All therapists were independently licensed master's‐level clinicians who were trained in using the CPT manual and had attended a 2‐day training session led by a CPT coinventor (Resick, LoSavio et al., 2024; Resick, Monson, & Chard, 2024). Therapists received intensive supervision on two CPT training cases to receive certification to see a full CPT caseload. Therapists also had access to daily CPT technique supervision and weekly consultation with the coinventor of CPT.

The following inclusion criteria were applied during data extraction: adult (18 years of age and older), clinical PTSD diagnosis at intake, and completed massed CPT between July 2023 and April 2025. Participants were excluded if they were under 18 years of age, did not have a clinical PTSD diagnosis at intake, and did not complete massed CPT during the aforementioned study period. Massed treatment was defined by an average of at least three sessions per week throughout treatment. Because this study is a retrospective analysis of secondary data, it was exempt from additional consent requirements under human subjects regulations and received approval from the Advarra Institutional Review Board (Protocol #Pro00077382). All study data were deidentified before analysis, and participants were not compensated for this study.

### Measures

#### Demographic characteristics

At intake, patients complete a demographic questionnaire that included questions about their age, sex, race, ethnicity, educational attainment level, and military service.

#### PTSD symptoms

The PTSD Checklist for *DSM‐5* (PCL‐5; Weathers et al., 2013) is a 20‐item, self‐report screening tool that assesses all 20 PTSD symptoms included in the *Diagnostic and Statistical Manual of Mental Disorders* (5th ed., text rev.; *DSM‐5‐TR*; American Psychiatric Association, 2022). The PCL‐5 has demonstrated strong internal consistency (i.e., Cronbach's αs = .94–.96), test–retest reliability (*r* = .82), and excellent convergent and structural validity across diverse samples (Blevins et al., 2015). Items are rated on a 5‐point Likert scale ranging from 0 (*not at all*) to 4 (*extremely*), with ratings summed and total possible scores of 0–80. Probable PTSD was defined as a total PCL‐5 score greater than 31 (Bovin et al., 2016). Minimal symptoms were defined as a score of 18 or lower, and mild symptoms were defined as scores of 19–30 (Forehand et al., 2022).

#### Depressive symptoms

The Patient Health Questionnaire–9 (PHQ‐9; Kroenke et al., 2001) is a nine‐item scale that measures symptoms of depression over the last 2 weeks. The PHQ‐9 has shown strong internal consistency (Cronbach's αs = .86–.89) and strong test–retest validity, with established construct and criterion validity for detecting depression symptoms. Respondents rated items on a 4‐point Likert scale ranging from 0 (*not at all*) to 3 (*nearly every day*), with item ratings summed and total possible scores of 0–27. Severity was defined as follows: minimal: 0–4, mild: 5–9, moderate: 10–14, moderately severe: 15–19, and severe: 20–27.

Of note, the Patient Health Questionnaire–8 (PHQ‐8; Kroenke et al., 2009) includes the same questions as the PHQ‐9, except for the ninth item, which assesses suicidal ideation. The PHQ‐9 was used to evaluate depressive symptom severity at intake and discharge, and the PHQ‐8 was used to evaluate depressive symptom severity at 30 and 90 days postdischarge. These measures are highly correlated and have similar diagnostic accuracy (Wu et al., 2020) and have been used interchangeably, as in our study (Wells et al., 2013).

#### Anxiety symptoms

The Generalized Anxiety Disorder–7 (GAD‐7; Spitzer et al., 2006) is a seven‐item scale that measures symptoms of generalized anxiety over the last 2 weeks. The GAD‐7 has demonstrated strong internal consistency (Cronbach's αs = .89–.92), test–retest reliability (*r* = .82), and strong convergent validity with other anxiety measures. Respondents rated items on a 4‐point Likert scale ranging from 0 (*not at all*) to 3 (*nearly every day*), with item ratings summed and total possible scores of 0–21. Severity was defined as follows: minimal: 0–4, mild: 5–9, moderate: 10–14, and severe: 15–21.

### Data analysis

Descriptive statistics for demographic, clinical, and treatment variables were calculated for the analytic sample (means and standard deviations for continuous variables, sample sizes and percentages for categorical variables). Although this analysis focused on individuals who participated in massed CPT, those who completed nonmassed treatment did not meaningfully differ on baseline characteristics (i.e., age; sex; race; ethnicity; and intake scores on measures of PTSD, depressive, and anxiety symptoms) from those who completed massed treatment (see Supplementary Table S1). Additionally, we examined differences between massed participants who completed the 30‐ and 90‐day postdischarge follow‐up assessments and those who did not complete postdischarge follow‐up assessments (Supplementary Table S2).

Paired samples *t* tests were used to evaluate average changes in PCL‐5, GAD‐7, and PHQ‐9 scores from baseline (intake) to discharge. Effect sizes were calculated using Cohen's *d* for paired samples to estimate the magnitude of the observed changes. Effect sizes were interpreted according to conventional benchmarks of 0.20, 0.50, and 0.80 for small, medium, and large effects, respectively (Cohen, 2013). We calculated the proportion of patients with probable PTSD (PCL‐5 score greater than 31; Bovin et al., 2016) and the proportion of patients who achieved reliable recovery (PCL‐5 score of 18 or below; Forehand et al., 2022). Due to variability in the timing of the postdischarge follow‐up assessments, observations were binned into 15–61 days for 30‐day follow‐up and 62–135 days for 90‐day follow‐up. The closest date to the ideal postdischarge follow‐up date (i.e., 30 or 90 days) was selected for the analysis. A sensitivity analysis model using a linear mixed‐effects model with continuous days since discharge confirmed that the binning approach did not bias the results (See Supplementary Table S3 and Supplementary Figure S1).

A treatment‐on‐the‐treated (TOT) analytic approach that focused on participants who received the intervention as intended (i.e., completing three sessions per week) was selected because the study objective was to evaluate the effectiveness of the massed CPT delivery format among individuals who engaged with this treatment format as designed rather than the overall impact of random assignment alone. To assess robustness, an intention‐to‐treat (ITT) analysis that included all enrolled participants regardless of treatment completion status was conducted (See Supplementary Table S4 and Supplementary Figure S2). Linear mixed‐effects models examined changes in symptom scores over time (intake, discharge, 30 days postdischarge, 90 days postdischarge) for each outcome (PCL‐5, GAD‐7, PHQ‐9). Time was entered as a categorical predictor with a random intercept to account for repeated measures within patients. Intake served as the reference category to estimate symptom change at each assessment point relative to pretreatment (i.e., baseline) symptom levels. Covariates (fixed effects) included age (centered), sex, race, ethnicity, and educational attainment level. For nondichotomous variables, the reference category was selected as the most common category endorsed in the sample. Models with a random effect of time were tested but did not converge due to limited postdischarge data. Degrees of freedom (*df*) and *p* values for fixed effects were computed using Satterthwaite's approximation (Satterthwaite, 1946), and parameters were estimated using restricted maximum likelihood. Post hoc pairwise comparisons between time points were conducted to evaluate the magnitude of change between discharge and subsequent postdischarge assessments. Bonferroni‐adjusted pairwise comparisons of estimated marginal means were examined to evaluate differences between time points. Data were analyzed using R (Version 2024.12.0+467; R Core Team, 2024). Linear mixed‐effects models were fit using the *lme4* package (Bates et al., 2015).

## RESULTS

### Demographic and descriptive statistics

**FIGURE 1:**
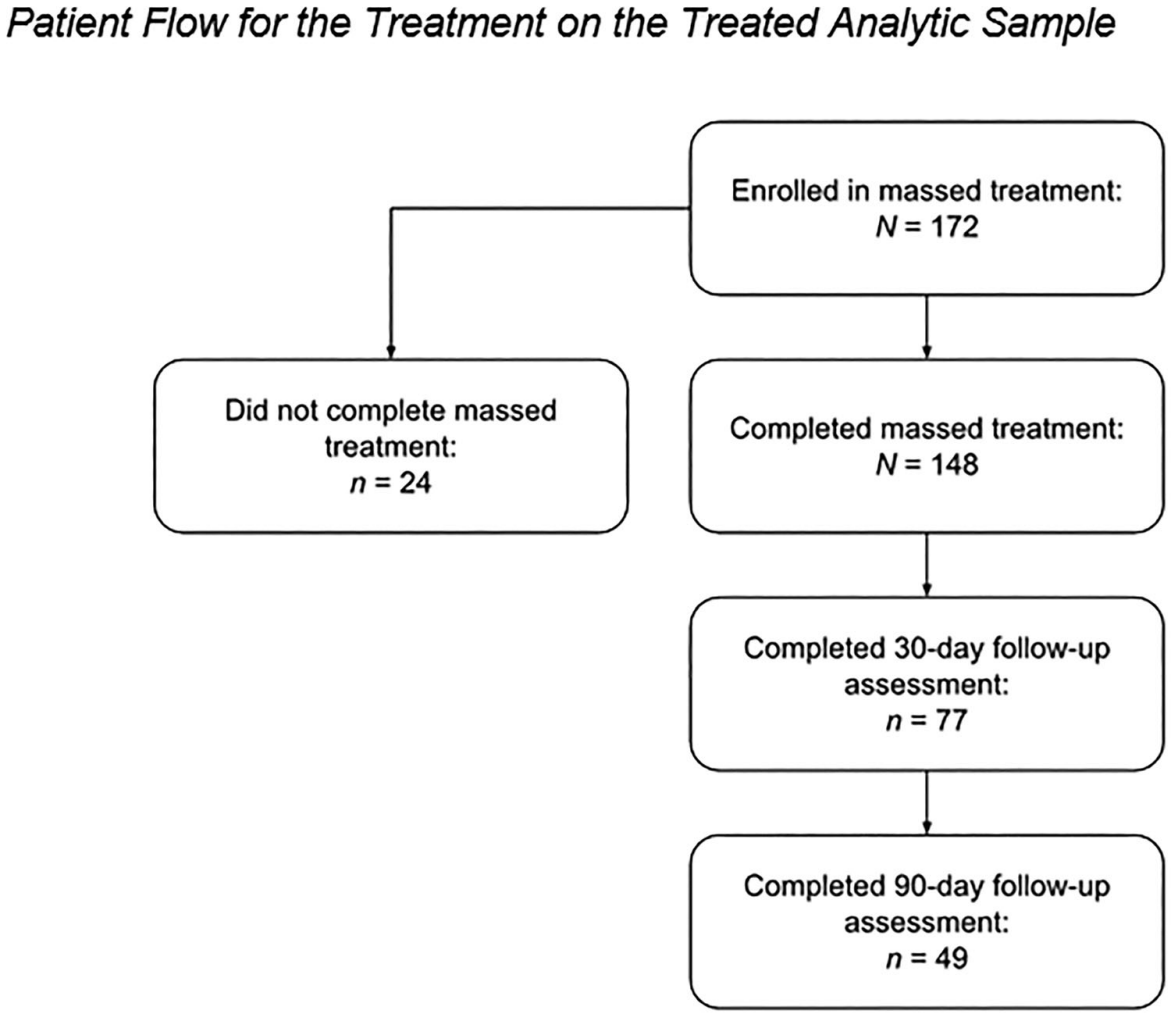
*Patient* *flow for the treated analytic sample*

A total of 148 patients completed massed treatment and were included in the analytic sample. Table 1 presents the demographic characteristics and trauma experiences of the patients, and Figure 1 presents participant flow for inclusion in the TOT analytic sample. Patients spent an average of 22.18 days (*SD* = 5.63) in treatment, completed an average of 3.73 massed CPT sessions per week (*SD* = 0.54), and completed an average of 12.43 sessions overall (*SD* = 1.98, *Mdn* = 12). Patients’ average intake scores on the PCL‐5 (*M* = 50.67, *SD* = 11.40), GAD‐7 (*M* = 14.66, *SD* = 4.77), and PHQ‐9 (*M* = 14.78, *SD* = 6.21) were clinically elevated. Patients’ average discharge scores on the PCL‐5 (*M* = 11.67, *SD* = 7.91), GAD‐7 (*M* = 4.76, *SD* = 4.10), and PHQ‐9 (*M* = 5.55, *SD* = 4.25) were all in the mild or minimal range. Sex was the only variable that differed significantly between patients who did and did not complete postdischarge follow‐up, with a higher proportion of male participants completing follow‐up assessments relative to female participants (See Supplementary Table S2).

**TABLE 1:** Demographic characteristics of the analytic sample

Variable	*M*	*SD*
Age at intake (years)	40.75	12.20

	*n*	%
Sex		
Female	123	83.1
Male	23	15.5
Missing	2	1.4
Race		
White	90	60.8
Missing	30	20.3
Black or African American	10	6.8
Multiracial	9	6.1
Asian	8	5.4
American Indian or Native	1	0.7
Ethnicity		
Not Hispanic or Latino	111	75.0
Hispanic or Latino	25	16.9
Missing	12	8.1
Educational attainment		
Bachelor's or associate's degree	66	44.6
Master's degree and above	50	33.8
High school or equivalent	25	16.9
Vocational certificate or training	5	3.4
Missing	2	1.4
Military service	1	0.7
Traumatic experiences^a^		
Childhood physical or sexual abuse	78	29.8
Intimate partner violence	49	18.7
Sexual assault	42	16.1
Grief and loss	20	7.6
Other	17	6.5
Physical violence	15	5.7
Serious accident or injury	15	5.7
Medical trauma	11	4.2
Pregnancy and/or birth complications	9	3.4
Transportation accident	4	1.5
Civilian gun violence	1	0.4
Natural disaster	1	0.4

*Note. N* = 148.

^a^ In the analytic sample, 32 participants reported trauma from a single event, 51 participants reported trauma from a series of events, and 65 participants reported trauma from both a single event and a series of events.

### Changes in PTSD, anxiety, and depressive symptoms between intake and discharge

The results of paired‐samples *t* tests comparing intake and discharge symptom scores revealed significant improvement in participants’ PTSD symptoms (PCL5: Δ*M* = 39.00, 95% confidence interval [CI; 36.84, 41.16]), *t*(147) = 35.75, *p* < .001; anxiety symptoms (GAD‐7: Δ*M* = 8.90, 95% CI [8.09, 9.71]), *t*(147) = 21.76, *p* <  .001; and depressive symptoms (PHQ‐9: Δ*M* = 8.23, 95% CI [7.33, 9.13]), *t*(147) = 18.07, *p* < .001. Difference scores were associated with large effect sizes for PTSD symptoms, *d* = 2.94, 95% CI [2.57, 3.31]; anxiety symptoms, *d* = 1.79, 95% CI [1.53, 2.05]; and depressive symptoms, *d* = 1.49, 95% CI [1.25, 1.72]. Of the 148 included patients, 146 scored below the clinical cutoff for probable PTSD at discharge (i.e., PCL‐5 score below 31; 98.6%), and 124 reported minimal symptom severity (i.e., of 18 or below; 83.8%).

### Changes in PTSD, anxiety, and depressive symptoms between intake, discharge, and postdischarge

**FIGURE 2:**
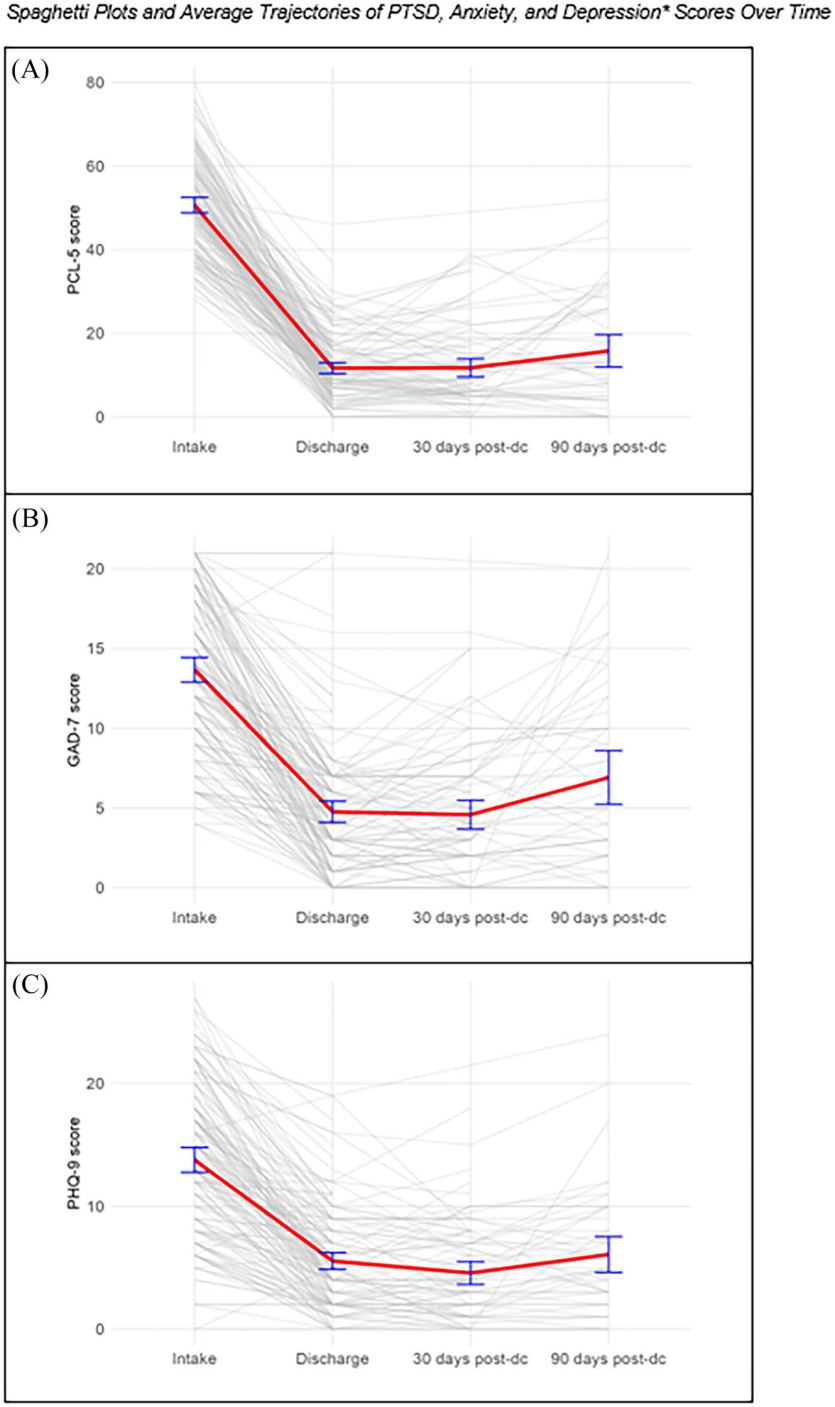
*Spaghetti plots and average trajectories for* *(A) posttraumatic stress disorder (PTSD)*, *(B) anxiety, and (C) depressive* *symptom scores over time* *Note*: The eight‐item version of the Patient Health Questionnaire (PHQ‐8) was used to measure depressive symptoms at the 30 and 90 days postdischarge follow‐up assessments rather than the nine‐item version (PHQ‐9), which was used at other assessment points. Gray lines represent individual participant trajectories. The red line indicates the sample mean at each time point, including intake (*N* = 148 for all measures), discharge (*N* = 148 for all measures), 30 days postdischarge (*n* = 77 for PTSD symptoms, PCL‐5, *n* = 72 for anxiety and depressive symptoms), and 90 days post‐discharge (*n* = 49 for PTSD and depressive symptoms, *n* = 48 for anxiety symptoms). Blue error bars represent 95% confidence intervals around the mean. Post‐dc = postdischarge; PCL‐5 = PTSD Checklist for *DSM‐5*; GAD‐7 = Generalized Anxiety Disorder–7.

Spaghetti plots of patient raw scores and mean trajectories of PTSD, anxiety, and depressive symptoms are presented in Figure 2. There was attrition at each time point beyond discharge. At 30 days postdischarge, 77 participants completed the PCL‐5, 72 completed the GAD‐7, and 72 completed the PHQ‐8. At 90 days postdischarge, 49 participants completed the PCL‐5, 49 completed the PHQ‐8, and 48 completed the GAD‐7. Pretreatment–posttreatment changes in PTSD, anxiety, and depressive symptoms did not predict follow‐up completion, indicating that attrition was not related to treatment response (See Supplementary Material). Findings from the linear mixed effects models are presented in Table 2. The pattern of results remained consistent when all participants who initiated massed treatment were included (i.e., ITT approach; see Supplementary Table S4 and Supplementary Figure S2).

**TABLE 2:** Fixed effects of linear mixed effects models for posttraumatic stress disorder (PTSD), anxiety, and depressive symptom outcome

	PCL‐5			GAD‐7			PHQ‐9^a^		
Variable	*B*	*SE*	*p*	*B*	*SE*	*p*	*B*	*SE*	*p*
Fixed effect^b^									
Intercept	50.82	3.15	< .001	13.23	1.63	< .001	15.43	1.85	< .001
Discharge	−39.10	1.17	< .001	−8.97	0.44	< .001	−8.49	0.47	< .001
30 days postdischarge	−39.87	1.44	< .001	−9.29	0.57	< .001	−9.74	0.60	< .001
90 days postdischarge	−35.46	1.70	< .001	−6.54	0.66	< .001	−7.85	0.70	< .001
Age (centered)	−0.09	0.06	.131	−0.06	0.03	.078	−0.04	0.04	.230
Sex (male)^c^	4.29	1.77	.018	1.61	0.94	.090	2.12	1.07	.051
Race^b^ (American Indian/Native)	−11.69	8.00	.147	−1.24	4.20	.769	−8.27	4.79	.087
Race (Asian)^c^	0.35	2.79	.901	−2.31	1.44	.111	−2.62	1.64	.113
Race^b^ (Black/African American)	1.59	2.47	.522	−0.55	1.30	.672	0.34	1.48	.820
Race^b^ (Multiracial)	−4.32	2.74	.118	−0.85	1.43	.552	−2.09	1.62	.201
Ethnicity (not Hispanic or Latino)	−0.87	3.05	.777	0.59	1.59	.712	−1.30	1.81	.472
Educational attainment (High school/equivalent)^c^	−0.29	1.95	.882	−1.59	1.03	.125	−0.38	1.17	.743
Educational attainment (Master's degree and above)^c^	0.95	1.62	.560	0.80	0.85	.349	−0.37	0.96	.704
Education (Vocational certificate/training)^c^	1.38	3.75	.713	−1.03	1.94	.598	1.23	2.17	.572

Random effect	Var.	*SD*		Var.	*SD*		Var.	*SD*	
Intercept	23.85	4.88		10.24	3.20		13.92	3.732	
Residual	80.73	8.99		11.36	3.37		12.85	3.585	

*Note. n* = 118 (of *N* = 148, due to missing demographic data). PCL‐5 = PTSD Checklist for *DSM‐5*; GAD‐7 = Generalized Anxiety Disorder–7; PHQ‐9 = Patient Health Questionnaire–9.

^a^ The eight‐item version of the Patient Health Questionnaire (i.e., PHQ‐8) was used to measure depressive symptoms at 30 and 90 days postdischarge rather than the nine‐item version (PHQ‐9), which was used at other assessment points.

^b^ Average outcome score at intake for patients in the reference categories and at the average age within the sample.

^c^ Reference groups were female (sex), White (race), and bachelor's or associate's degree (educational attainment).

*p < .05. ***p < .001.

The model‐estimated PCL‐5 intercept was 50.82, *p* < .001. PCL‐5 scores significantly decreased at discharge, *B =* −39.10, *p* < .001; 30 days postdischarge, *B =* −39.87, *p* < .001; and 90 days post‐discharge, B = −35.46, *p* < .001, compared to intake. Male participants experienced significantly more PTSD symptoms on average than female participants, regardless of time point, *B* = 4.29, *p* = .018. Post hoc pairwise comparisons indicated no significant change in PCL‐5 scores between discharge and 30 days postdischarge, *B* = .76, *p* = 1.00; between 30 days postdischarge and 90 days postdischarge, *B* = −4.40, *p* = .120; or between discharge and 90 days postdischarge, *B* = −3.64, *p* = .199.

The model‐estimated GAD‐7 intercept was 13.23, *p* < .001. GAD‐7 scores significantly decreased at discharge, *B* = −8.97, *p* < .001; 30 days postdischarge, *B* = −9.28, *p* < .001; and 90 days postdischarge, *B =* −6.54, *p* < .001, compared to intake. No covariates were significantly associated with GAD‐7 outcomes. Post hoc pairwise comparisons indicated no significant change in GAD‐7 scores between discharge and 30 days postdischarge, *B =* .31, *p* = 1.00; a slight increase between 30 and 90 days postdischarge, *B =* −2.74, *p* < .001; and a slight increase between discharge and 90 days postdischarge, *B =* −2.43, *p* < .001.

The model‐estimated PHQ‐9 intercept was 15.43, *p* < .001. PHQ‐9 scores significantly decreased at discharge, *B =* −8.49, *p* < .001; 30 days postdischarge, *B =* −9.74, *p* < .001; and 90 days postdischarge, *B =* −7.85, *p* < .001, compared to intake. No covariates were significantly associated with PHQ‐9 outcomes. Post hoc pairwise comparisons indicated no significant change between PHQ‐9 scores at discharge and PHQ‐8 scores at 30 days postdischarge, *B =* 1.25, *p* = .240; no significant change in PHQ‐8 scores between 30 and 90 days postdischarge, *B =* −1.89, *p* = .090; and no significant change in PHQ‐8 scores between PHQ‐9 scores at discharge and PHQ‐8 scores at 90 days postdischarge, *B =* −.64, *p* = 1.00.

## DISCUSSION

The purpose of this study was to evaluate changes in PTSD, anxiety, and depressive symptoms over the course of virtual massed CPT delivered by a commercial virtual trauma clinic. We aimed to (a) test whether PTSD, anxiety, and depressive symptom scores significantly improved from intake to discharge; (b) examine symptom trajectories across intake, discharge, and 30 and 90 days postdischarge; and (c) determine whether symptom improvements observed at discharge were maintained at 30 and 90 days postdischarge. PTSD, anxiety, and depressive symptoms decreased significantly between intake and discharge, and these improvements were sustained up to 90 days postdischarge.

Patients showed significant improvements in PTSD symptoms from intake to discharge with a large effect size, *d* = 2.94, exceeding effect sizes reported in previous studies. For example, when comparing nonmassed CPT to a control group, meta‐analytic findings showed a mean between‐group Hedges’ *g* of 1.24 (Asmundson et al., 2019). Individual trials of massed CPT with veterans (Goetter et al., 2021; Yamokoski et al., 2023) and civilians (Held et al., 2022) reported within‐group Cohen's *d* effect sizes ranging from 1.8 to 2.5. Our effect size was substantially larger than the within‐group effect size reported in the only published study of a commercial virtual clinic delivering blended care therapy for PTSD symptoms (*d* = 1.48; Owusu et al., 2025). Our observed effect size in a civilian, commercially delivered format lies at the upper end of previously reported effect sizes across populations and delivery contexts. At discharge, almost all patients in our sample (approximately 99%) had PCL‐5 scores below the threshold for probable PTSD (i.e., above 31), and most (approximately 84%) scored in the minimal symptom range (i.e., 18 or lower). The robust clinical response rates observed in this study may be explained by our use of both variable and massed treatment dosing. Variable treatment dosing (i.e., flexibly adjusting the number of sessions based on patient needs and progress) can increase CPT response rates (Galovski et al., 2012). Although most participants completed the standard number of sessions typically observed in CPT for PTSD (12 sessions [Resick, LoSavio, et al., 2024]; *M* = 12, *Mdn* = 12, range: 5–17), the ability to tailor the number of sessions based on individual progress may have supported retention and maximized therapeutic gains, particularly within the massed delivery format. This flexible yet intensive approach may have helped individuals achieve an optimal therapeutic dose.

Participants experienced significant improvements in depressive and anxiety symptoms from intake to discharge. The large effect sizes observed for improvement in depression (*d* = 1.49) and anxiety (*d* = 1.79) are consistent with prior research on traditional CPT formats (i.e., weekly, in‐person; Resick et al., 2012) and massed CPT (Held et al., 2022). Our findings support the transdiagnostic benefits of CPT delivered in a massed format and virtual setting and suggest that virtual massed CPT efficiently targets multiple symptom domains with a single treatment protocol rather than requiring separate interventions for each condition or an explicitly transdiagnostic protocol. As prior research has established the efficacy of standard, in‐person CPT to improve comorbid symptoms (e.g., insomnia, suicidal behaviors, and substance use; LoSavio et al., 2024), future studies may build on the present findings by exploring whether virtual massed CPT yields similar transdiagnostic benefits to standard CPT in community and civilian samples (Najavits et al., 2020; Zalta et al., 2020).

PTSD symptom improvements were maintained at both 30 and 90 days postdischarge in this sample. Although emerging evidence suggests that massed CPT results in sustained improvements in PTSD symptoms, this research has been limited to small community or predominantly veteran samples (Goetter et al., 2021; Held et al., 2022). The current study provides important, real‐world evidence of PTSD symptom maintenance in a broader population receiving virtual massed CPT. These sustained improvements align with CPT's emphasis on teaching individuals transferable cognitive and emotional skills they can apply independently following treatment completion (Resick, Monson, & Chard, 2024). Future research should examine factors associated with long‐term maintenance of treatment gains, such as patient characteristics, treatment dose and duration, treatment process variables (e.g., cognitive processes, therapeutic alliance), and skill retention and generalization (i.e., applying skills learned in therapy to situations outside of therapy) postdischarge.

Depressive symptoms followed a similar maintenance pattern as PTSD symptoms, with no significant changes through 90 days postdischarge. Generalized anxiety symptoms showed a slight but significant increase between 30 and 90 days postdischarge, though this was still a large overall improvement from baseline. The sustained depressive symptom improvements align with controlled research on massed CPT and other intensive PTSD treatments (Held et al., 2022, 2020; Oprel et al., 2021) and extend these findings to a real‐world setting. The modest anxiety rebound indicates that generalized anxiety may require additional maintenance strategies to prevent symptom recurrence. Understanding these differential maintenance patterns is important for identifying which patients may benefit from enhanced posttreatment support. Future research should explore the types of posttreatment support that best mitigate anxiety symptom recurrence.

This study has several strengths. To our knowledge, this study is the largest real‐world evaluation of virtual massed CPT to date (i.e., *N* > 100 participants). The relatively large, naturalistic sample of nonveteran patients with a PTSD diagnosis increases the findings’ generalizability to treatment‐seeking civilian populations in community settings. The observational design allowed us to examine treatment outcomes under real‐world conditions, adding evidence for the effectiveness of massed CPT to existing efficacy research. This study measured outcomes beyond primary PTSD symptoms, including depressive and anxiety symptoms, enhancing the ability to understand the effects of virtual massed CPT on highly comorbid conditions. Finally, the inclusion of postdischarge follow‐up assessments allowed us to understand the durability of treatment gains following the termination of intensive treatment.

This study also has limitations. Our primarily White and highly educated sample limits generalizability to more diverse groups. CPT is effective across a variety of settings and communities, including lower‐literacy and international populations (Bass et al., 2013; Holliday et al., 2017). Future research should confirm whether the virtual, massed delivery format of CPT is as accessible and effective across diverse settings and communities as traditional CPT. This retrospective, observational study lacked a control or comparison group by design, limiting our ability to attribute symptom improvements to massed CPT alone or to other aspects of the treatment program. This study did not assess process variables, differences in trauma types, and potential mechanisms of change (e.g., treatment engagement, therapeutic alliance, homework, trauma cognitions) that could meaningfully enhance understanding of how and why massed CPT works. The limited sample at both postdischarge time points, which was due to attrition, may have influenced the observed maintenance of treatment improvements. Although telehealth can expand access to care for individuals in less‐affluent or underserved communities, the findings from this study may be less generalizable to populations without reliable access to commercial digital health platforms. Together, patient differences in demographic characteristics, accessibility, and socioeconomic factors may all influence CPT engagement and outcomes. Future research should include larger, more diverse samples and explore treatment mechanisms and outcomes over a longer follow‐up period. Additionally, future studies should determine the optimal number of massed CPT sessions and identify which patients benefit from different treatment doses.

Patients in this sample showed significant decreases in PTSD, depressive, and anxiety symptoms over the course of massed CPT. Improvements were largely maintained over time, indicating the durable effects of these rapid treatment gains. The results highlight the clinical impact and feasibility of virtual massed CPT for treating PTSD and comorbid symptoms. Our findings underscore the promise of specialized virtual trauma clinics to deliver accessible, evidence‐based mental health care to a wide range of individuals affected by trauma. Future research should examine implementation factors that contribute to successful virtual massed CPT delivery, including the feasibility of the virtual platform (e.g., usability and satisfaction), treatment fidelity, therapist training protocols, and specialized supervision practices.

## AUTHOR NOTE

Eric Lin, Lashauna Cutts, Sofia Noori, Isobel Rosenthal, and Kathleen Chard are employed by Nema Health, and Nema Health was the behavioral health treatment used in this study. Debra Kaysen is a paid consultant of Nema Health, employed by Stanford University, and this work was not part of her Stanford duties and responsibilities. Jennifer Huberty is a paid consultant of Nema Health. Jacqlyn Yourell and Courtney Jewell are employed by Jennifer Huberty. Authors’ employment status and salary are not dependent upon the results of their research. Lara Baez has no conflicts of interest to declare.

## OPEN PRACTICES STATEMENT

The dataset used for this retrospective analysis is not publicly available, given Nema Health's privacy policy for patient data. Aggregated and anonymized data may be shared with interested parties upon reasonable request at jackie@fit-minded.com.

## AUTHOR CONTRIBUTIONS

Lara Baez: writing ‐ original draft, methodology, writing ‐ review and editing, formal analysis. Jennifer Huberty: conceptualization, writing ‐ original draft, writing ‐ review and editing, methodology, supervision. Jacqlyn Yourell: writing ‐ review and editing, writing ‐ original draft, methodology, supervision. Courtney Jewell: writing ‐ review and editing, formal analysis, methodology. Eric Lin: data curation, supervision, formal analysis, writing ‐ review and editing, methodology. Debra Kaysen: writing ‐ review and editing, conceptualization. Lashauna Cutts: conceptualization, writing ‐ review and editing. Sofia Noori: conceptualization, writing ‐ review and editing. Isobel Rosenthal: conceptualization, methodology, writing ‐ review and editing, supervision. Kathleen Chard: conceptualization, writing ‐ review and editing.

## Supporting information




**Table S1** Descriptive Statistics of Demographic, Baseline, and Treatment Characteristics in the Non‐Massed and Massed Samples
**Table S2** Descriptive Statistics of Demographic, Baseline, and Treatment Characteristics Across Post‐Discharge Follow‐up Completion Groups
**Table S3**. Linear Mixed Effects Models Sensitivity Analysis
**Table S4**. Fixed Effects of Linear Mixed Effects Models for PTSD, Anxiety, and Depression Outcomes (n = 135)
**Figure S1**. Sensitivity Analysis: Estimated Symptom Change
**Figure S2**. Spaghetti Plots and Average Trajectories of PTSD, Anxiety, and Depression* Scores Over Time
