# Supplementary material for: Effectiveness of massed cognitive processing therapy for posttraumatic stress disorder: A retrospective analysis
Source: J Trauma Stress. 2026 Jan 19;39(2):307–18. doi: 10.1002/jts.70045 (PMC13044385; doi:10.1002/jts.70045)
Supplement: Supplementary file 1 — Table S1 Descriptive Statistics of Demographic, Baseline, and Treatment Characteristics in the Non‐Massed and Massed Samples Table S2 Descriptive Statistics of Demographic, Baseline, and Treatment Characteristics Across Post‐Discharge Follow‐up Completion Groups Table S3. Linear Mixed Effects Models Sensitivity Analysis Table S4. Fixed Effects of Linear Mixed Effects Models for PTSD, Anxiety, and Depression Outcomes (n = 135) Figure S1. Sensitivity Analysis: Estimated Symptom Change Figure S2. Spaghetti Plots and Average Trajectories of PTSD, Anxiety, and Depression* Scores Over Time [file JTS-39-307-s001.docx]

Effectiveness of Massed Cognitive Processing Therapy for Posttraumatic Stress Disorder: A Retrospective Analysis

Supplemental Results

*Comparisons of Demographic and Treatment Characteristics between Massed and Non-Massed Samples*

Independent t-tests for continuous variables and chi-square tests for categorical variables were used to evaluate differences in baseline characteristics between the non-massed (*n* = 147) and massed (*N* = 148) samples. Most demographic characteristics were similar across groups. Education level did significantly differ between the massed and non-massed groups. This is because the non-massed group has more patients with high school education and fewer patients with a masters degree or higher. We do not expect this to meaningfully impact our results, given there is no established meaningful association between education level and massed treatment outcomes.

Treatment characteristics associated with massed treatment, such as time in treatment and average weekly sessions, differed between groups as expected. Patients in the massed treatment group completed treatment in less time and had more weekly sessions. However, groups did not differ on the number of sessions attended. Baseline clinical characteristics (i.e., PCL-5, GAD-7, and PHQ-9) did not significantly differ between the groups.

**Table S1**

*Descriptive Statistics of Demographic, Baseline, and Treatment Characteristics in the Non-Massed and Massed Samples*

|  | Non-massed sample  (*n* = 147) | | Massed sample  (*N* = 148) | | Test statistic | *p* |
| --- | --- | --- | --- | --- | --- | --- |
|  | *n* | % | *n* | % |  |  |
| Sex | | | | | | |
| Female | 117 | 79.6 | 123 | 83.1 | *χ^2^*(*df* = 1) = .78 | .377 |
| Male | 30 | 20.4 | 23 | 15.5 |  |  |
| Missing | 0 | 0.0 | 2 | 1.4 |  |  |
| Race | | | | | | |
| Other | 4 | 2.7 | 0 | 0.0 | Fisher’s Exact *p* | .404 |
| American  Indian or  Native | 0 | 0.0 | 1 | 0.7 |  |  |
| Asian | 8 | 5.4 | 8 | 5.4 |  |  |
| Black or  African  American | 9 | 6.1 | 10 | 6.8 |  |  |
| Multiracial | 7 | 4.8 | 9 | 6.1 |  |  |
| White | 91 | 61.9 | 90 | 60.8 |  |  |
| Missing | 28 | 19.0 | 30 | 20.3 |  |  |
| Ethnicity | | | | | | |
| Hispanic or  Latino | 22 | 15.0 | 25 | 16.9 | *χ^2^*(*df* = 1) = .044 | .835 |
| Not Hispanic  or Latino | 110 | 74.8 | 111 | 75.0 |  |  |
| Missing | 15 | 10.2 | 12 | 8.1 |  |  |
| Education | | | | | | |
| Bachelor’s or  associate’s  degree | 65 | 44.2 | 66 | 44.6 | Fisher’s Exact *p* | .042 |
| High school  or equivalent | 39 | 26.5 | 25 | 16.9 |  |  |
| Master’s  degree and  above | 33 | 22.4 | 50 | 33.8 |  |  |
| Vocational  certificate or  training | 10 | 6.8 | 5 | 3.4 |  |  |
| Missing | 0 | 0.0 | 2 | 1.4 |  |  |
| Military | 5 | 3.4 | 1 | 0.7 | Fisher’s Exact *p* | .214 |
|  |  |  |  |  |  |  |
|  | *M* | *SD* | *M* | *SD* |  |  |
| Age at intake | 40.12 | 13.75 | 40.65 | 12.49 | *t*(285.47) = -.43 | .671 |
| Days in Treatment | 36.79 | 14.19 | 22.18 | 5.63 | *t*(190.65) = 11.61 | <.001 |
| Average Sessions Per Week | 2.36 | 0.45 | 3.73 | 0.54 | *t*(274.07) = -23.79 | <.001 |
| Total Number of Sessions | 12.32 | 2.69 | 12.43 | 1.98 | *t*(268.17) = -0.39 | .700 |
| Intake PCL-5 | 50.41 | 12.88 | 50.67 | 12.88 | *t*(288.27) = -0.18 | .859 |
| Intake GAD-7 | 13.80 | 5.15 | 13.66 | 5.15 | *t*(290.97) = 0.24 | .808 |
| Intake PHQ-9 | 14.67 | 6.35 | 13.78 | 6.21 | *t*(292.75) = 1.22 | .225 |

*Note.* Mean (*SD*) is reported for continuous variables. Categorical variables are reported as *n* (%). *t*-tests were used to compare continuous variables and chi-square or Fisher’s exact tests (for variables with cells with small *N*s) for categorical variables.

*Comparisons of Demographic and Treatment Characteristics Between Massed Completers Who Completed Post-Discharge Follow-up Assessments and Massed Completers Who Did Not Complete Post-Discharge Follow-up Assessments.*

Differences between participants who completed massed treatment (*N* = 148) who completed post-discharge follow-up assessments at 30- and/or 90-days with those who did not complete post-discharge follow-up assessments were examined. Participants were categorized into three mutually exclusive groups based on post-discharge follow-up assessment completion: participants who completed no follow-up assessments (*n* = 60), participants who completed a 30-day follow up assessment (*n* = 39), and participants who completed a 90-day follow-up assessment.

Most demographic and treatment variables did not significantly differ across post-discharge follow-up groups. The only significant difference was observed for sex, as there was a higher proportion of males in the 30-day follow-up group compared to those with no follow-up. Specifically, the cohort that did not attend any follow-up assessments included a greater proportion of females (94.9%) compared with the 30-day only cohort (74.4%, adjusted *p* = 0.025), however, there was a small sub-sample of males who completed the 30- and 90-day follow up assessments. No other demographic, treatment, or clinical variables differed significantly between groups, suggesting that post-discharge follow-up cohorts were comparable.

Lastly, we evaluated whether clinical improvement influenced the likelihood of completing post-discharge follow-up assessments. PCL-5 symptom improvement ($\Delta$PCL-5 OR = 0.83, 95% CI [0.46-1.45], *p* = .515), PHQ symptom improvement ($\Delta$PHQ OR = 1.06, 95% CI [0.65-1.74], *p* = .807), and GAD symptom improvement ($\Delta$GAD-7 OR = 1.35, 95% CI [0.88-2.13], *p* = .173) were unrelated to the completion of follow-up assessments. These findings suggest that missing follow-up assessments were not systematically associated with symptom improvement from treatment.

**Table S2**

*Descriptive Statistics of Demographic, Baseline, and Treatment Characteristics Across Post-Discharge Follow-up Completion Groups*

|  | No post-discharge follow-up assessments completed  (*n* = 60) | | Completed 30-day follow-up assessment  (*n* = 39) | | Completed 90-day follow-up assessment  (*n* = 49) | | *p* |
| --- | --- | --- | --- | --- | --- | --- | --- |
|  | *n* | *%* | *n* | *%* | *n* | *%* |  |
| Sex | | | | | | | |
| Female | 56 | 93.3 | 29 | 74.0 | 38 | 77.5 | .007 |
| Male | 3 | 5.0 | 10 | 26.0 | 10 | 20.4 |  |
| Missing | 1 | 1.7 | 0 | 0.0 | 1 | 2.1 |  |
| Race | | | | | | | |
| American  Indian or  Native | 0 | 0.0 | 1 | 2.6 | 0 | 0.0 | 0.871 |
| Asian | 4 | 6.7 | 2 | 5.1 | 2 | 4.1 |  |
| Black or  African  American | 2 | 3.3 | 4 | 10.3 | 4 | 8.2 |  |
| Multiracial | 4 | 6.7 | 2 | 5.1 | 3 | 6.1 |  |
| White | 33 | 55.0 | 25 | 64.1 | 32 | 65.3 |  |
| Missing | 17 | 28.3 | 5 | 12.8 | 8 | 16.3 |  |
| Ethnicity | | | | | | | |
| Hispanic or  Latino | 13 | 21.7 | 4 | 10.3 | 8 | 16.3 | 0.249 |
| Not Hispanic  or Latino | 40 | 66.7 | 33 | 84.6 | 38 | 77.5 |  |
| Missing | 7 | 11.6 | 2 | 5.1 | 3 | 6.2 |  |
| Education | | | | | | | |
| Bachelor’s or  associate’s  degree | 22 | 36.7 | 18 | 46.2 | 26 | 53.1 | .481 |
| High school or  equivalent | 10 | 16.7 | 7 | 17.9 | 8 | 16.3 |  |
| Master’s  degree and  above | 24 | 40.0 | 12 | 30.8 | 14 | 28.6 |  |
| Vocational  certificate or  training | 3 | 5.0 | 2 | 5.1 | 0 | 0.0 |  |
| Missing | 1 | 1.0 | 0 | 0.0 | 1 | 2.0 |  |
| Military* | 0 | 0.0 | 1 | 2.6 | 0 | 0.0 | .267 |
|  | *M* | *SD* | *M* | *SD* | *M* | *SD* |  |
| Age at intake | 40.28 | 12.62 | 39.69 | 11.99 | 42.18 | 11.94 | .597 |
| Days in treatment | 21.97 | 5.82 | 22.89 | 4.96 | 21.85 | 5.95 | .647 |
| Intake PCL-5 | 51.31 | 11.36 | 50.28 | 11.99 | 50.18 | 11.16 | .851 |
| Intake GAD-7 | 13.32 | 5.07 | 14.15 | 4.52 | 13.69 | 4.64 | .696 |
| Intake PHQ-9 | 13.66 | 5.89 | 15.28 | 6.56 | 12.71 | 6.18 | .154 |

*Note. *n* (%) of participants that reported yes to military service. ANOVAs were used for continuous variables and Fisher’s exact tests were used for categorical variables. Participants were grouped by the following post-discharge follow-up assessment completion status: no post-discharge follow-up assessments completed (*n* = 60), 30-day only (*n* = 39), or 90-day (*n* = 49); total *N* = 148.

*Sensitivity analysis of post-discharge follow-up binning using days since discharge.*

Linear mixed effects models were fit using days since discharge as a continuous variable to examine the robustness of using predefined 30- and 90-day bins. Table S3 and Figure S1 present findings. These findings mirror the direction and magnitude of the main analyses, suggesting that the binned time approach (15 - 61, and 62 - 135) did not bias results.

**Table S3.**

*Linear Mixed Effects Models Sensitivity Analysis*

| Outcome | Estimate | *SE* | *t* |
| --- | --- | --- | --- |
| PCL-5 | 0.04 | 0.01 | 3.59 |
| PHQ | 0.01 | 0.00 | 2.60 |
| GAD-7 | 0.02 | 0.01 | 4.48 |

**Figure S1.**

*Sensitivity Analysis: Estimated Symptom Change*

| *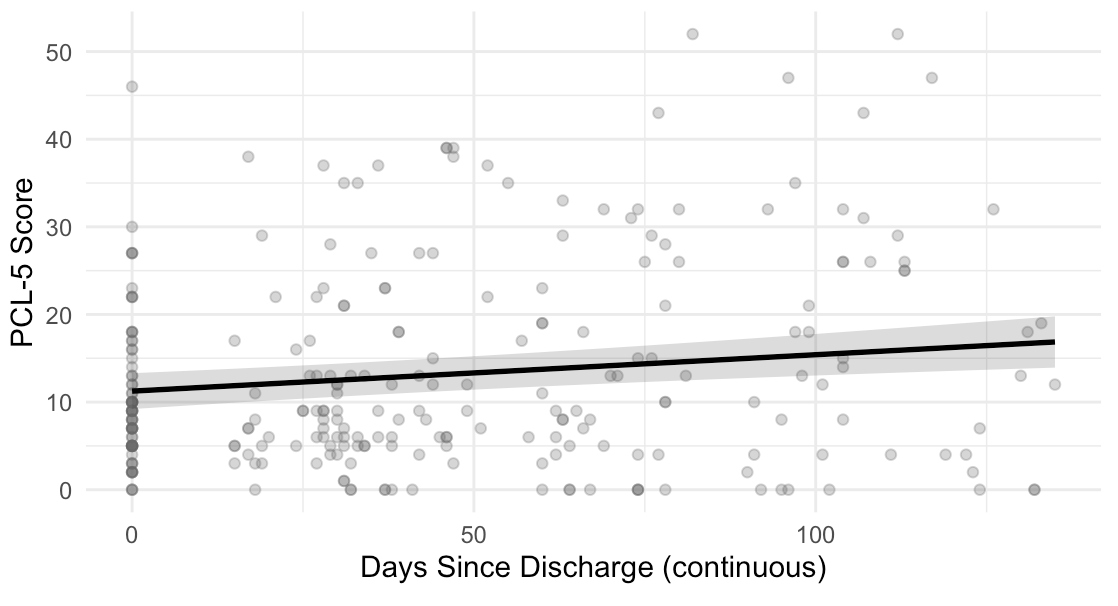* |
| --- |
| *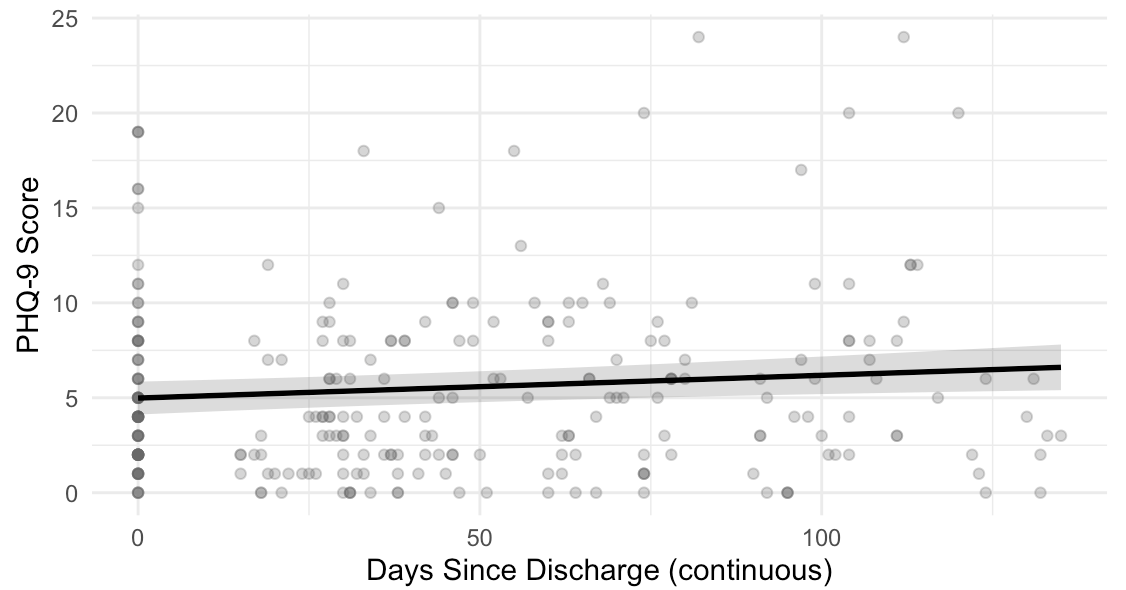* |
| *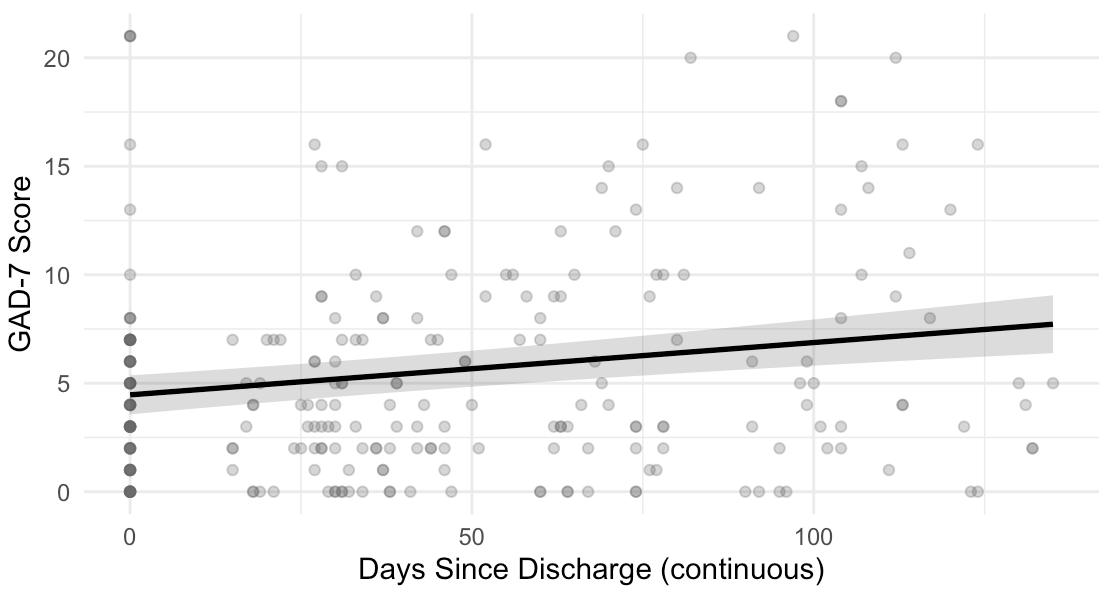* |

*Changes in PTSD, Anxiety, and Depression Symptoms Between Intake, Discharge, and Post-Discharge (Intention to Treat Analysis)*

To determine whether linear mixed-effects models were robust to the effects of dropout, we conducted a secondary analysis that included all participants who enrolled in the massed treatment, regardless of whether they completed the full treatment. Participants were included in this model if they completed at least 3 sessions of CPT per week; however, they were not required to complete all scheduled treatment. This model does not include individuals who participated in non-massed treatment (i.e., less than 3 sessions of CPT per week).

**Table S4.**

*Fixed Effects of Linear Mixed Effects Models for PTSD, Anxiety, and Depression Outcomes (n = 135)*

|  | PCL-5 | | | | GAD-7 | | | | PHQ-9* | | | |
| --- | --- | --- | --- | --- | --- | --- | --- | --- | --- | --- | --- | --- |
| Fixed Effect: | *β* | *SE* | | *p* | *β* | *SE* | | *p* | *β* | *SE* | | *p* |
| Intercept | 38.55 | 9.95 | | < .001 | 12.12 | 1.80 | | < .001 | 14.40 | 2.05 | | < .001 |
| Discharge | -35.96 | 1.28 | | < .001 | -8.27 | 0.43 | | < .001 | -8.03 | 0.44 | | < .001 |
| 30 Days Post-Discharge | -39.33 | 1.67 | | < .001 | -9.14 | 0.58 | | < .001 | -9.70 | 0.61 | | < .001 |
| 90 Days Post-Discharge | -34.95 | 1.99 | | < .001 | -6.39 | 0.68 | | < .001 | -7.79 | 0.71 | | < .001 |
| Age (centered) | -0.12 | 0.07 | | .104 | -0.06 | 0.03 | | .065 | -0.07 | 0.04 | | .065 |
| Sex (Male) | 2.42 | 2.29 | | .293 | 0.98 | 1.03 | | .341 | 1.43 | 1.17 | | .225 |
| Race (Asian) | 8.28 | 11.11 | | .458 | -2.57 | 1.53 | | .096 | -2.59 | 1.75 | | .142 |
| Race (Black or African American) | 13.29 | 10.74 | | .219 | 0.58 | 1.30 | | .659 | 1.17 | 1.49 | | .434 |
| Race (Multiracial) | 4.61 | 10.72 | | .668 | -0.28 | 1.50 | | .850 | -1.41 | 1.72 | | .413 |
| Ethnicity (Not Hispanic or Latino) | 2.44 | 3.97 | | .540 | 1.86 | 1.76 | | .292 | 0.07 | 2.01 | | .972 |
| Education(High school or equivalent) | 0.48 | 2.41 | | .842 | -1.13 | 1.07 | | .291 | 0.37 | 1.22 | | .763 |
| Education (Master’s degree and above) | 2.51 | 2.03 | | .218 | 1.29 | 0.90 | | .154 | 0.38 | 1.03 | | .712 |
| Education (Vocational certificate or training) | 6.30 | 4.18 | | .134 | 0.35 | 1.83 | | .471 | 2.59 | 2.07 | | .213 |
|  | PCL-5 | | | | GAD-7 | | | | PHQ-9* | | | |
| Random effect: | Variance | | *SD* | | Variance | | *SD* | | Variance | | *SD* | |
| Intercept | 52.55 | | 7.25 | | 13.69 | | 8.70 | | 18.92 | | 4.35 | |
| Residual | 110.42 | | 10.51 | | 12.33 | | 3.51 | | 13.34 | | 3.65 | |

*Note.* Linear mixed effects models included participants from the massed treatment cohort (*n* = 172) who had available data across at least two study timepoints. Participants without valid repeated-measure outcome data (*n* = 37) were excluded from this linear mixed-effects model. Reference groups: Female, White, Bachelor’s or Associate’s Degree.

*SE* = Standard Error

*PHQ-8 was used to measure depression symptoms at 30 and 90 days post-discharge

**Figure S2.**

*Spaghetti Plots and Average Trajectories of PTSD, Anxiety, and Depression* Scores Over Time*

| **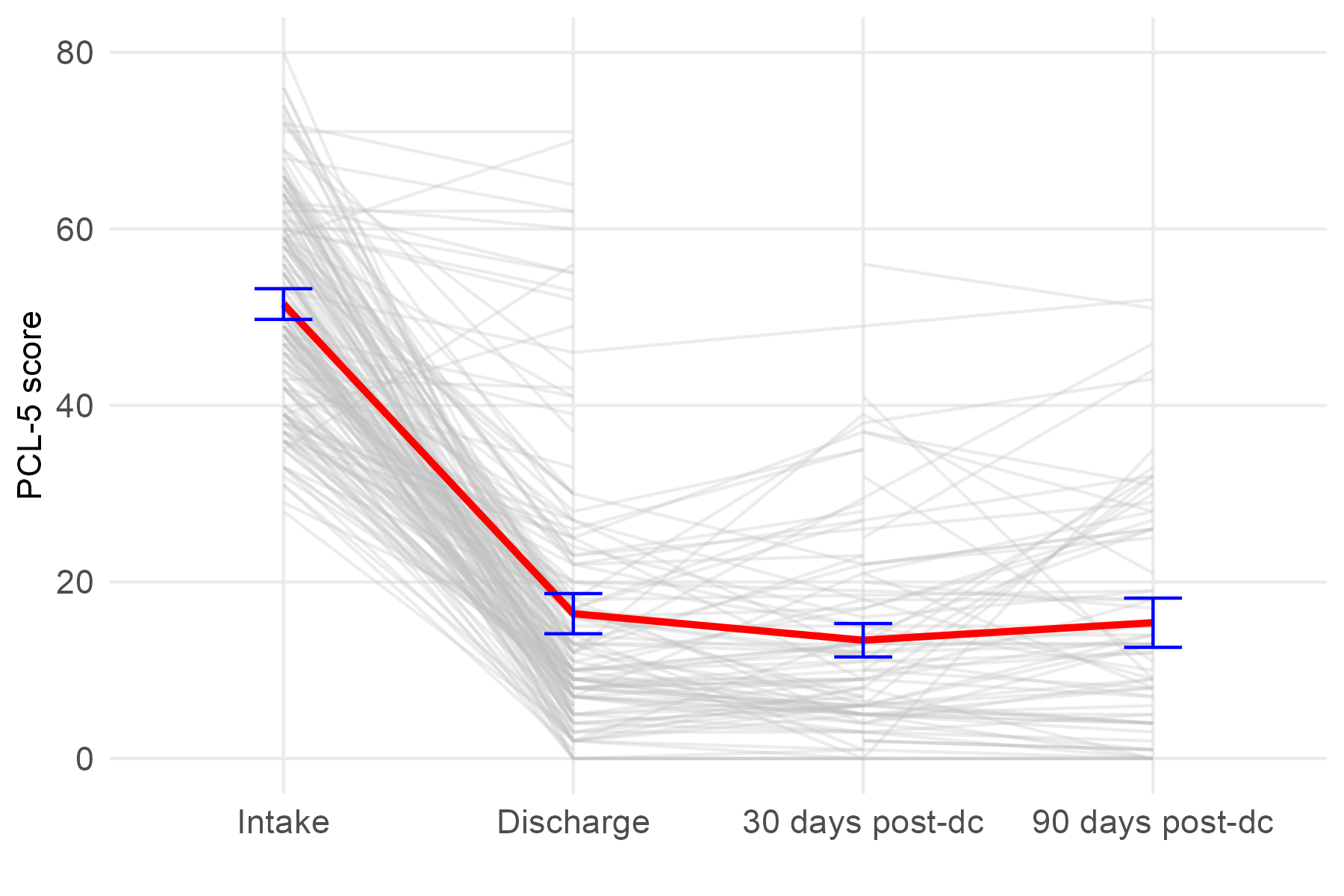** |
| --- |
| **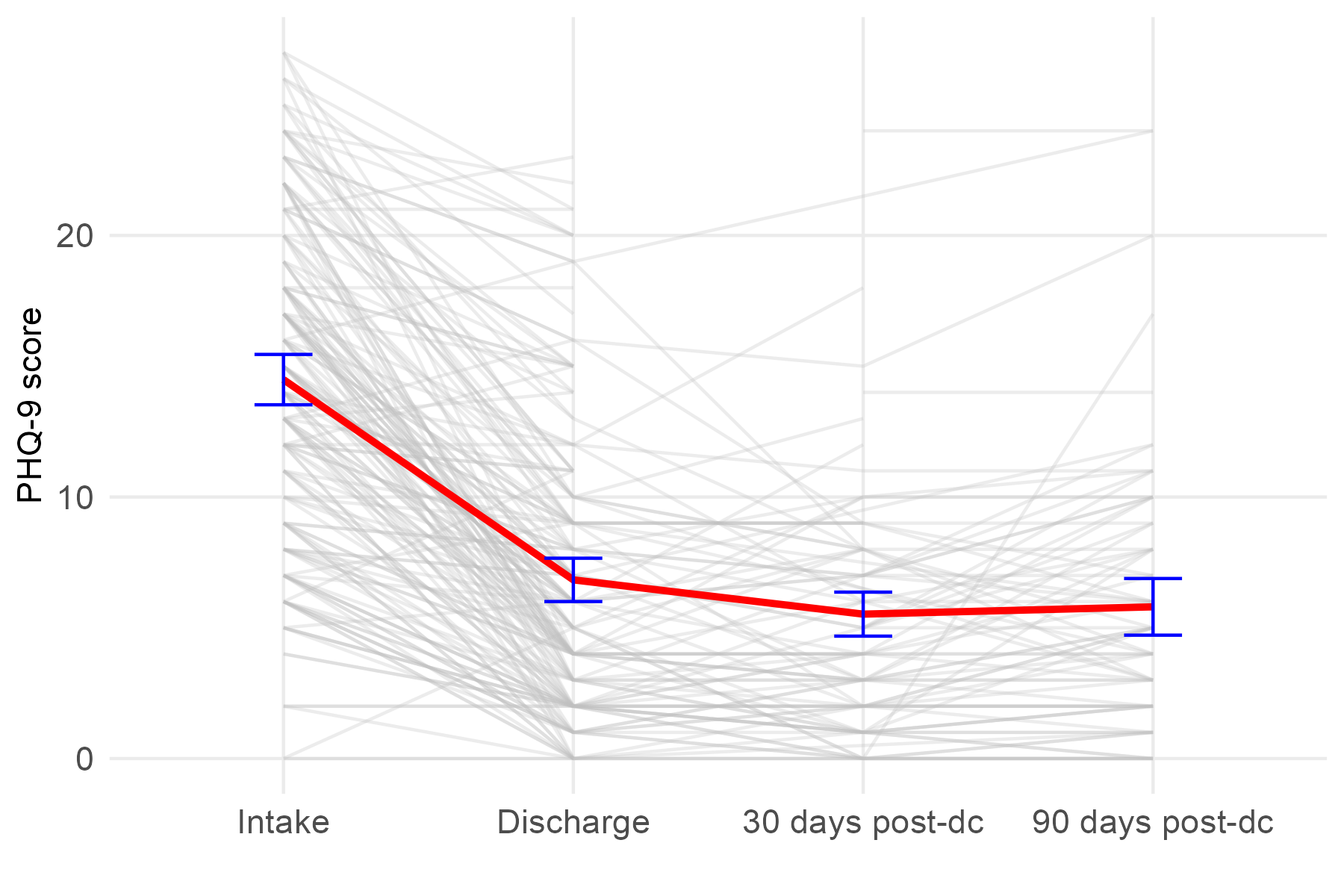** |
| **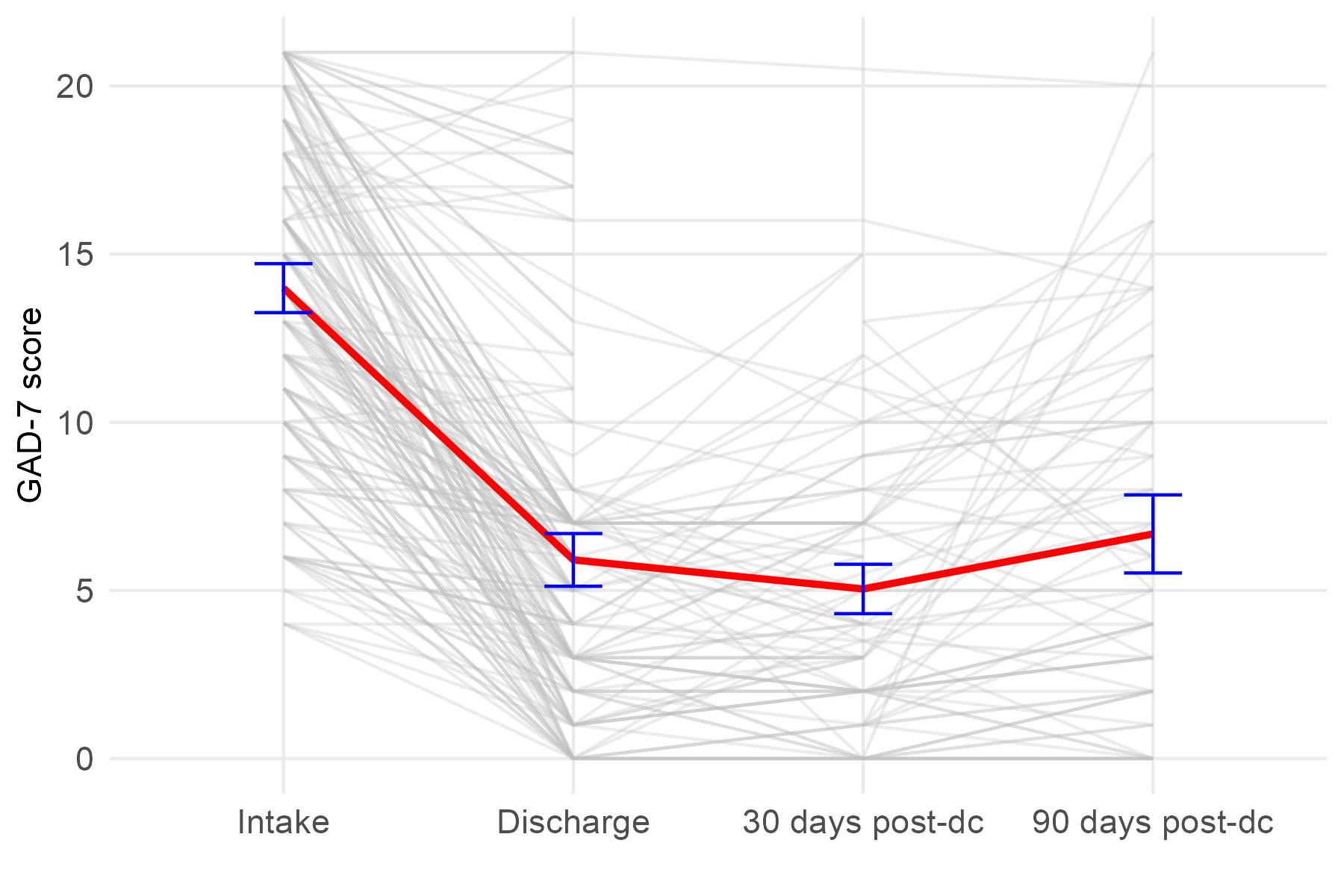** |
